
JOURNAL INFORMATION
==============================
NLM Title Abbreviation: Schizophr Bull
Journal ID: Schizophr Bull
NLM Journal ID: 0236760
Journal ID: schbul

Schizophrenia Bulletin

ISSN: 0586-7614
EISSN: 1745-1701
Publisher: Oxford University Press

ARTICLE INFORMATION
==============================
PMCID: PMC5887492
DOI: 10.1093/schbul/sby016.310
Article ID: sby016.310
Article version: 1
Subjects: Abstracts, Poster Session I

T34. Submission Withdrawn

Print publication date: 2018 Apr
Electronic publication date: 2018 Apr 1
Volume: 44
Issue: Suppl 1
Issue title: Abstracts for the Sixth Biennial SIRS Conference
First page: S126
Last page: S126
Copyright: © Maryland Psychiatric Research Center 2018.
Copyright year: 2018
License: This is an Open Access article distributed under the terms of the Creative Commons Attribution License (http://creativecommons.org/licenses/by/4.0/), which permits unrestricted reuse, distribution, and reproduction in any medium, provided the original work is properly cited.
License URL: https://creativecommons.org/licenses/by/4.0/

Page count: 1

==============================
### T34. SUBMISSION WITHDRAWN .

### T35. DIFFUSION MEASURES OF EXTRACELLULAR FREE WATER IN A NON-HUMAN PRIMATE MODEL OF MATERNAL IMMUNE ACTIVATION: EXPLORING NEUROIMMUNE MECHANISMS OF PSYCHIATRIC DISORDERS

Cameron Carter*[,1], Tyler Lesh[2], Costin Tanase[2], Jeffrey Bennett[2], Ana-Maria Iosif[2], Judy Van Der Water[2], Richard Maddock[2], David Amaral[2], Melissa Baumann[2]
*[1]UC Davis Health System, Imaging Research Center; [2]UC Davis*

**Background:** Evidence has been accumulating for an immune-based component of psychiatric disorder etiology, particularly schizophrenia. Early epidemiological studies found an increased incidence of schizophrenia in offspring of mothers who had an infection during pregnancy. Recent work has identified genetic links to the MHC complex, pro-inflammatory cytokine elevations in cerebrospinal fluid and plasma. We have developed a non-human primate (NHP) model of maternal immune activation (MIA) using a modified form of the viral mimic polyIC (polyICLC) examine the relationship between altered neuorimmune function may contribute to psychosis risk through effects on the developing brain and behavior of NHP offspring. In a previous cohort of MIA-exposed offspring, our group observed evidence for increased pre-synaptic dopamine levels in the striatum using 6-[18F]fluoro-L-m-tyrosine (FMT) positron emission tomography, in addition to pubertal-onset behavioral abnormalities, which may model part of the neurodevelopmental pathway towards psychosis. This study builds on this model and examines the effect of maternal immune activation on in vivo--extracellular free water--a diffusion magnetic resonance imaging measure obtained with a multi-shell acquisition. We sought to test the hypothesis that offspring of pregnant monkeys who received polyICLC injections at the end of the first trimester would show increased extracellular free water compared to control offspring.

**Methods:** Fourteen pregnant rhesus monkeys (Macaca mulatta) receiving polyICLC at the end of the first trimester were compared to 14 controls. The offspring from both groups underwent a multi-shell diffusion MRI scan at 3 Tesla. Diffusion data was collected when the offspring were one month, 6 months, and 12 months of age. Six month preliminary findings are currently presented. Diffusion images were aligned to individual subject MPRAGE scans. Individual subject structural scans were then nonlinearly aligned to generate a common group average template and the group average template was subsequently nonlinearly aligned to a neurodevelopmental rhesus atlas. For this preliminary analysis, the frontal cortex was selected as an a priori region of interest in addition to the more global whole-brain gray and white matter masks.

**Results:** Six month old MIA-exposed rhesus offspring showed a trend for increased whole-brain white matter extracellular free water ($p=.09$) with no significant difference in whole-brain gray matter free water ($p=.27$) compared to control offspring. However, analysis of the frontal ROI revealed significantly increased gray matter free water in the left hemisphere ($p=.013$) with a trend towards increased gray matter free water in the right hemisphere ($p=.081$). There were no significant differences between MIA-exposed and control offspring in basic motor and reflex development or growth trajectories.

**Discussion:** These data suggest that despite the lack of behavioral abnormalities at this early age, extracellular free water values are increased in MIA-exposed offspring, particularly in frontal gray matter. More global whole-brain free water group differences did not reach statistical significance, which may indicate some regional specificity to these changes early in development. The NHP MIA model complements the human schizophrenia literature in which extracellular free water increases have been repeatedly identified. Ultimately, these data provide validation of the clinical relevance of the NHP MIA model and improve our understanding of neuroimmune mechanisms in the development of psychiatric disorders, particularly schizophrenia.
